# Spontaneous development of Alzheimer's disease‐associated brain pathology in a Shugoshin‐1 mouse cohesinopathy model

**DOI:** 10.1111/acel.12797

**Published:** 2018-06-25

**Authors:** Chinthalapally V. Rao, Mudassir Farooqui, Yuting Zhang, Adam S. Asch, Hiroshi Y. Yamada

**Affiliations:** ^1^ Center for Cancer Prevention and Drug Development Department of Medicine Hematology/Oncology Section University of Oklahoma Health Sciences Center (OUHSC) Oklahoma City Oklahoma; ^2^ Stephenson Cancer Center Department of Medicine Hematology/Oncology Section University of Oklahoma Health Sciences Center (OUHSC) Oklahoma City Oklahoma

**Keywords:** amyloid beta, cohesinopathy, genomic instability, late‐onset Alzheimer's disease, mouse model, Shugoshin‐1 (Sgo1)

## Abstract

Spontaneous late‐onset Alzheimer's disease (LOAD) accounts for more than 95% of all human AD. As mice do not normally develop AD and as understanding on molecular processes leading to spontaneous LOAD has been insufficient to successfully model LOAD in mouse, no mouse model for LOAD has been available. Existing mouse AD models are all early‐onset AD (EOAD) models that rely on forcible expression of AD‐associated protein(s), which may not recapitulate prerequisites for spontaneous LOAD. This limitation in AD modeling may contribute to the high failure rate of AD drugs in clinical trials. In this study, we hypothesized that genomic instability facilitates development of LOAD and tested two genomic instability mice models in the brain pathology at the old age. Shugoshin‐1 (Sgo1) haploinsufficient (∓) mice, a model of chromosome instability (CIN) with chromosomal and centrosomal cohesinopathy, spontaneously exhibited a major feature of AD pathology; amyloid beta accumulation that colocalized with phosphorylated Tau, beta‐secretase 1 (BACE), and mitotic marker phospho‐Histone H3 (p‐H3) in the brain. Another CIN model, spindle checkpoint‐defective BubR1^−/+^ haploinsufficient mice, did not exhibit the pathology at the same age, suggesting the prolonged mitosis‐origin of the AD pathology. RNA‐seq identified ten differentially expressed genes, among which seven genes have indicated association with AD pathology or neuronal functions (e.g., ARC, EBF3). Thus, the model represents a novel model that recapitulates spontaneous LOAD pathology in mouse. The Sgo1^−/+^ mouse may serve as a novel tool for investigating mechanisms of spontaneous progression of LOAD pathology, for early diagnosis markers, and for drug development.

## 1 INTRODUCTION

Alzheimer's disease (AD) is a leading cause of cognitive impairment and death among people older than 65. Five percent of AD develops early in life (familial/early‐onset AD [EOAD]) facilitated by mutations in Amyloid Precursor Protein (APP), presenilin 1, Tau, or APOE genes. The remaining 95% of human AD is late onset (LOAD). The exact cause of LOAD remains unclear, although inflammation, oxidative stress, cholesterol metabolism, glycation, and other environmental and lifestyle factors are recognized as aggravating factors (Scheltens et al., 2016).

Major pathological features of the human AD brain include plaques of amyloid‐β made of cleaved APP, tangles of Tau proteins, and congophilic cerebral amyloid angiopathy (Kitazawa, Medeiros, & Laferl, 2012; Onos, Sukoff Rizzo, Howell, & Sasner, 2016; Sasaguri et al., 2016; Scheltens et al., 2016). With insufficient knowledge on the cause, modeling LOAD in rodents has been an issue. Normally, mice do not develop AD, which has been interpreted as due to their shorter lifespan and sequence differences in APP and Tau. Various transgenic mouse models with modified APP, Tau, and others have been developed for EOAD (Kitazawa et al., 2012; Onos et al., 2016; Sasaguri et al., 2016). However, LOAD models are limited to apes and are practically nonexistent in rodents. AD drugs developed with EOAD models were explored for use as human LOAD therapy, under an assumption that drugs effective on EOAD would also be effective in treating LOAD. More than 98% of drugs tested in EOAD rodent models were ineffective in human LOAD patients in clinical trials (Cummings, Morstorf, & Zhong, 2014), raising concerns on current drug targets and on the validity of the EOAD models for human LOAD.

Genomic instability and aneuploidy have been suspected to cause or aggravate AD, as high rates of both are present in the human AD brain (Bajic, Spremo‐Potparevic, Zivkovic, Isenovic, & Arendt, 2015). Further, genomic instability biomarkers have been associated with mild cognitive impairment and AD (Lee, Thomas, & Fenech, 2015). Aneuploidy may facilitate development of AD‐like dementia, as 15% of patients with Down syndrome with chromosome 21 trisomy develop AD‐like cognitive dysfunction in their 40s, and the rate increases to 50%–70% by age 60 (Bajic et al., 2015; Potter, 1991). However, a causal link between CIN, aneuploidy, and AD has not been established.

Genomic instability and aneuploidy have also been suspected to cause cancers (Boveri, 2008). To investigate the effects of genomic instability and resulting aneuploidy on carcinogenesis, various mouse models have been developed, mainly by targeting mitotic or other cell cycle regulators (Foijer, Draviam, & Sorger, 2008; Rao, Yamada, Yao, & Dai, 2009; Ricke, van Ree, & van Deursen, 2008; Schvartzman, Sotillo, & Benezra, 2010). Shugoshin‐1 (Sgo1) protects cohesin proteins and centrosome integrity (Salic, Waters, & Mitchison, 2004; Schöckel, Möckel, Mayer, Boos, & Stemmann, 2011). Cohesins keep sister chromatids from prematurely separating during mitosis, thus ensuring mitotic fidelity (Hirano, 2015). The Sgo1 haploinsufficient (∓) mouse is a model of cohesinopathy and chromosome instability (CIN) (Rao et al., 2016; Wang et al., 2008; Yamada et al., 2012, 2015, 2016). Sgo1^−/+^ model has shown unique transcriptomic signatures at the tissue/organ level and cancer proneness in certain organs including colon, lung, and liver (Rao et al., 2016; Yamada et al., 2012, 2015, 2016). In humans, the homolog SgoL1 is frequently mutated or abnormally expressed in cancers, affecting the mitotic process (Iwaizumi et al., 2009; Kahyo et al., 2011; Matsuura et al., 2013; Wang et al., 2015). Congenital mutations in human SgoL1 lead to chronic atrial and intestinal dysrhythmia syndrome, affecting the heart and gut rhythm (Chetaille et al., 2014). However, whether the mutations affect AD is unknown. The CAID syndrome is extremely rare disease. Chetaille et al. (2014) originally reported only 17 patients. The disease‐associated SgoL1 missense mutation was found in <1% in public database. A likely reason for the rarity of CAID syndrome is that congenital mutation in human SgoL1 may not be compatible with early development. In mouse, Sgo1 is highly expressed in heart, gut, and CNS during early development (Song et al., 2017a, 2017b), and Sgo1^−/−^ (knockout) is embryonic lethal (Yamada et al., 2012); strongly suggesting essential function of Sgo1 and SgoL1 during early development. Also, this study is the first ever study linking Sgo1 and AD. As such, no study focusing on correlation between the CAID syndrome and AD has been performed thus far.

BubR1 is a mitotic spindle checkpoint component. BubR1^−/+^ mice showed mitotic slippage in the cells and were colon cancer‐prone (Dai et al., 2004; Rao et al., 2005), and BubR1^H/H^ hypomorphic mice were identified as a model for premature aging (Baker et al., 2004). Neuronal cell division and axon growth were inhibited by siRNA‐mediated BubR1 knockdown in the mouse brain (Yang et al., 2017). The above findings in BubR1 transgenic models led to a hypothesis that mitotic errors and CIN facilitate AD‐like neurodegeneration.

With the current gap in LOAD study models, we hypothesized that LOAD development is facilitated by genomic instability with CIN. We tested whether the Sgo1^−/+^ or BubR1^−/+^ haploinsufficient mouse could serve as a model for spontaneous LOAD progression.

## 2 RESULTS

### 2.1 Middle‐aged Sgo1^−/+^ expressed Amyloid Beta Precursor Protein‐Binding Family B Member 1 (APBB1) at a higher amount in the whole blood

To identify biomarkers for CIN and cohesinopathy in whole blood RNA, we performed comparative whole blood RNA‐seq analysis on 12‐month‐old Sgo1^−/+^ and wild‐type mice. Among differentially expressed genes (*p *<* *0.05), Amyloid Beta Precursor Protein‐Binding Family B Member 1 (APBB1) was notable, with a 3.63‐fold increase compared with wild‐type control (Supporting Information Figure S1). APBB1 encodes a protein involved in DNA damage repair, interacts with APP, and is thought to promote AD. The pilot result at a younger age led us to suspect that the brains of Sgo1^−/+^ mice would show signs of neurodegenerative disease similar to AD.

### 2.2 Sgo1^−/+^ brains accumulated amyloid‐β by 24 months of age

**Figure 1:**
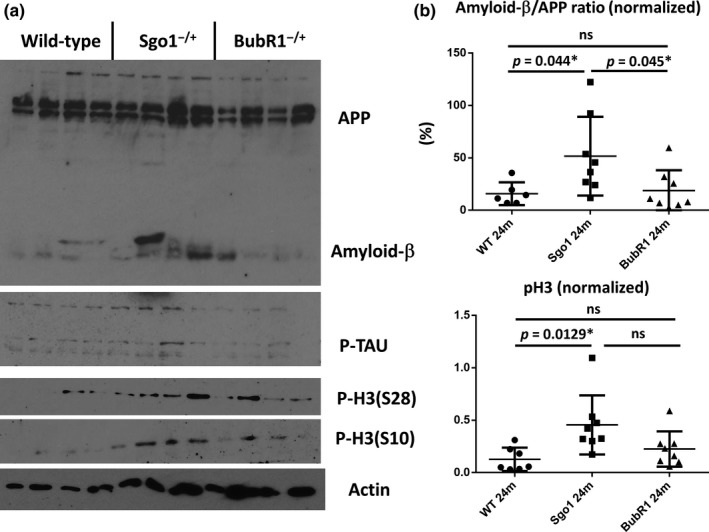
Sgo1^−/+^ brains accumulated amyloid‐β and mitotic marker phospho‐Histone H3 (pH3) by the 24‐month age equivalent to human old age. (a) Immunoblots for APP/amyloid‐β, p‐TAU, pH3 (Ser10), pH3 (Ser28), and β‐actin (loading control) in wild‐type (control), Sgo1^−/+^, and BubR1^−/+^ brain (cerebrum) extracts. (b) Amyloid‐β/APP ratio indicated significant accumulation of amyloid‐β only in the Sgo1^−/+^ brain. pH3 is most increased in Sgo1^−/+^ mice, compared with WT and BubR1^−/+^ mice

An aging‐and‐carcinogenesis study cohort provided Sgo1^−/+^ brains at older ages (24–25 months) corresponding to human old age over 65. We also collected brains from BubR1^−/+^ haploinsufficient mice to determine whether they show brain aging and AD pathology, as the initial hypothesis was focusing on CIN and AD. Increased accumulations of amyloid‐β (i.e., increase in amyloid‐β/APP ratio) were observed in brain extracts from Sgo1^−/+^ mice, but not from control littermate wild‐type or BubR1^−/+^ mice (Figure 1a,b). There was no significant difference in the total amount of phosphorylated TAU (Figure 1a).

With the assumption that CIN would affect AD pathology, we were puzzled by the result that our haploinsufficient BubR1^−/+^ model did not show more amyloid‐β in the brain than did wild‐type mice. A major difference between the Sgo1^−/+^ cohesinopathy model and the BubR1^−/+^ mitotic checkpoint‐defective model is the mitotic checkpoint function and existence (or absence) of prolonged mitosis. Sgo1^−/+^ brains showed higher expression of mitotic marker phospho‐Histone H3 (p‐H3), consistent with prolonged mitosis, while BubR1^−/+^ brains did not (Figure 1a,b).

### 2.3 Mitotic marker phosphorylated Histone H3‐positive cells are enriched with amyloid‐β, p‐TAU, and BACE (beta‐secretase 1)

**Figure 2:**
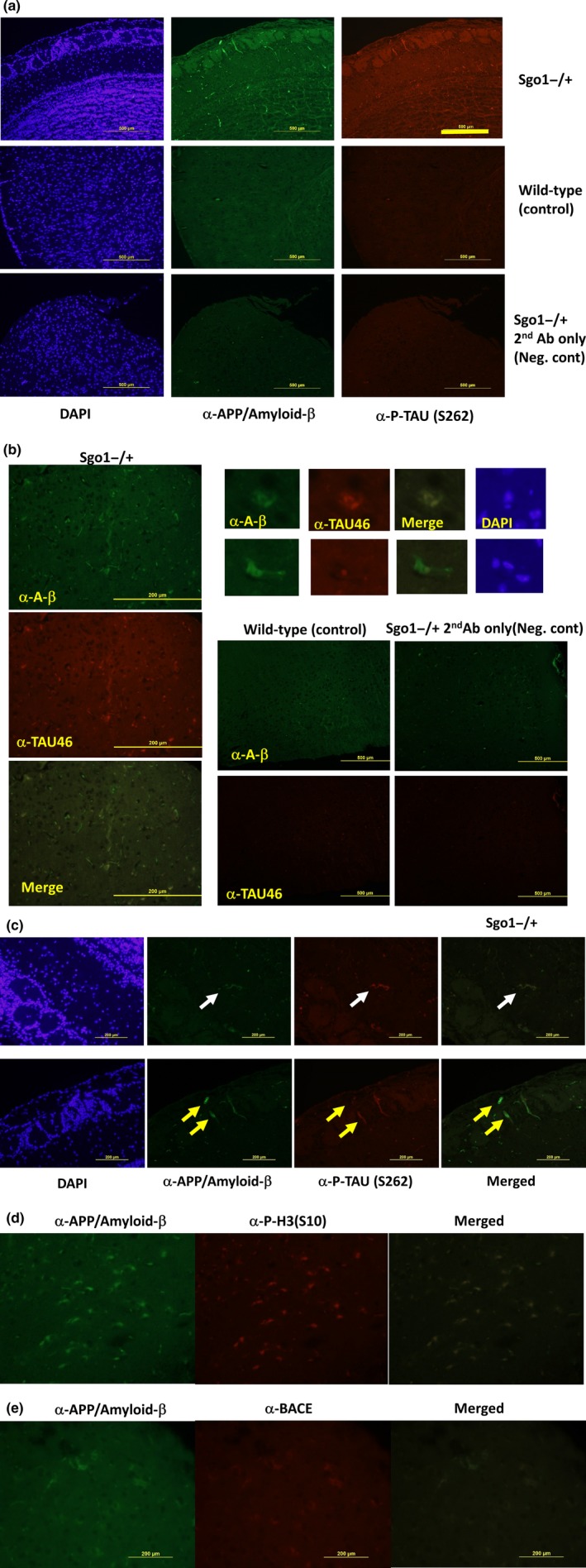
Sgo1^−/+^ brains displayed AD‐associated pathology that may originate from mitotic cells (a) Sgo1^−/+^ brain shows cells with APP/amyloid‐β and phosphorylated TAU. [Bar = 500 μm]. (b) Sgo1^−/+^ brain shows cells with amyloid‐β and TAU. [Bar = 200 μm]. Although they generally colocalize and appear in vicinity, they do not always show exact colocalization (enlarged panels). Staining controls (wild type or Sgo1^−/+^ without primary antibodies) provided much darker staining at the same image acquisition settings, except background signals from mouse IgG‐expressing‐infiltrating cells. [Bar = 500 μm]. (c) APP/amyloid‐β and phosphorylated TAU colocalized and appeared in extracellular matrix as a deposit (upper panel, white arrow) or in cells (lower panel, yellow arrows). (d) APP/amyloid‐β‐positive cells or deposits are also positive for mitotic marker phosphorylated Histone H3. (e) APP/amyloid‐β‐positive cells or deposits are also positive for BACE. [(c–e): Bar = 200 μm]

Immunofluorescence in Sgo1^−/+^ mice indicated that APP/amyloid‐β and TAU generally colocalized (Figure 2a: phosphorylated TAU; Figure 2b: TAU) and appeared in two forms: (a) extracellular deposits; and (b) cytoplasmic staining enriched in living cells. In addition to amyloid‐β deposits with p‐TAU (Figure 2c, upper panel), living cells coexpressing amyloid‐β and p‐TAU were observed (Figure 2c, lower panel). These findings suggest that the source of amyloid‐β/p‐TAU deposits may be live cells accumulating both.

To explain the difference between Sgo1^−/+^ and BubR1^−/+^ models, we next hypothesized that prolonged mitotic arrest is the trigger for amyloid‐β accumulation. The APP/amyloid‐β‐expressing cells were positive for mitotic marker p‐H3 (Figure 2D) and beta‐secretase 1 (BACE), an APP/amyloid‐β conversion enzyme (Figure 2e). The immunofluorescence results support the hypothesis that the source of accumulation of amyloid‐β is p‐H3‐positive cells that also coexpress BACE and p‐TAU. Overall, the results suggested that amyloid‐β and p‐TAU originated from p‐H3‐positive (prolonged) mitotic cells.

Next, we tested Congo red staining for amyloidosis, which did not provide clear staining in the Sgo1^−/+^ (not shown). Lack of Congo red staining suggested that degree of amyloid accumulation is not as high in this Sgo1^−/+^ model as existing EOAD mouse models that typically express a few‐to‐several‐fold amount of total amyloids compared with controls and show Congo red staining (Kitazawa et al., 2012; Onos et al., 2016; Sasaguri et al., 2016). The result was in agreement with immunoblots in Figure 1 indicating only mild increase in total amyloids (amyloid‐β and APP combined) in Sgo1^−/+^ compared with age‐matched wild type and BubR1^−/+^. The modest increase in total amyloids suggests that the Sgo1^−/+^ model may recapitulate relatively early phase of spontaneous LOAD development.

### 2.4 p‐H3 expression in Sgo1 was observed both in the cortex and in the hippocampus, while p‐H3 expression is limited to the hippocampus in wild type

**Figure 3:**
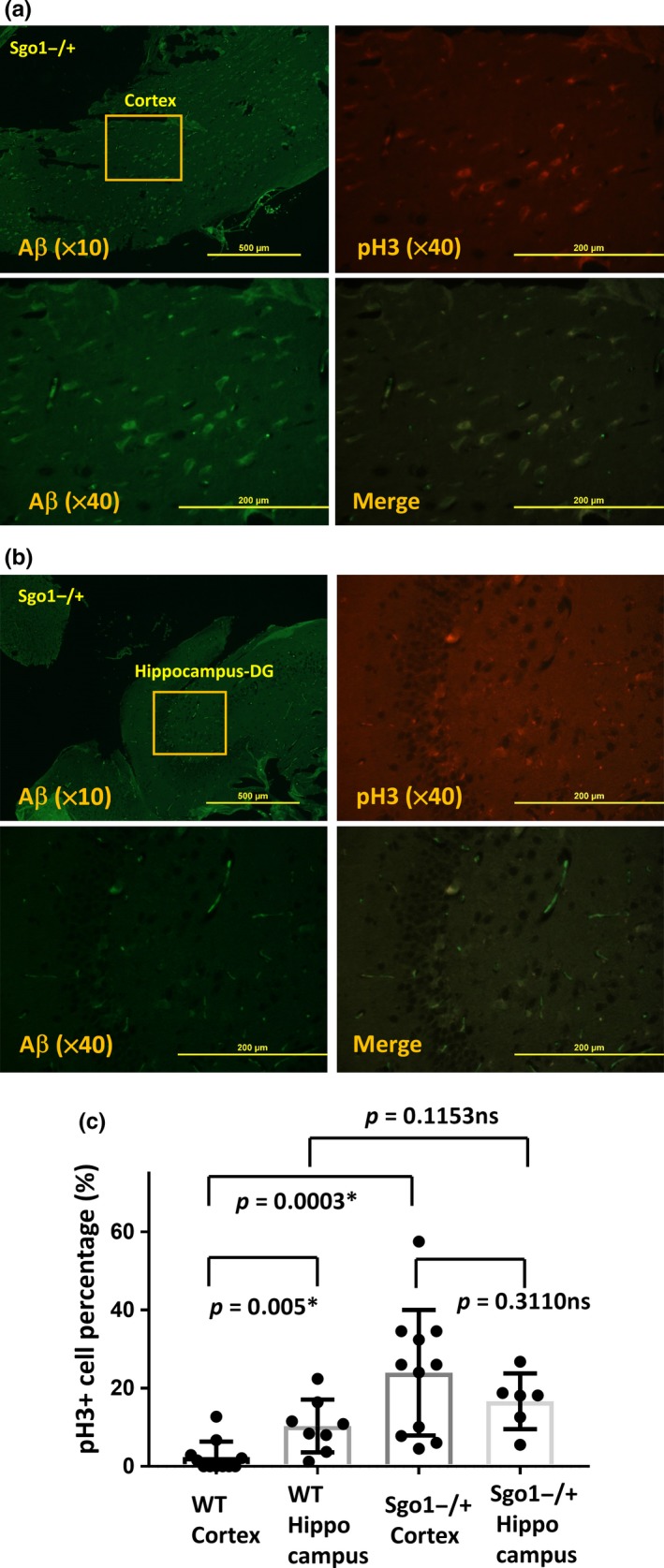
Sgo1^−/+^ brains accumulated amyloid‐β‐and‐p‐H3‐positive cells both in cortex and hippocampus, while in wild‐type p‐H3‐positive cells exclusively located in hippocampus. (a) Amyloid‐β‐and‐p‐H3‐positive cells locate in the cortex of Sgo1^−/+^. Cortex of Sgo1^−/+^ was stained with amyloid‐β, p‐H3, and DAPI. Upper‐left panel shows amyloid‐β staining in a lower magnification (×10) [Bar = 500 μm]. The squared area is presented in a higher magnification (×40) in the other panels for amyloid‐β (Aβ), p‐H3, and merge. [Bar = 200 μm]. (b) Amyloid‐β‐and‐p‐H3‐positive cells also locate in the hippocampus of Sgo1^−/+^. (c) Quantification of p‐H3‐positive cells in wild type and in Sgo1^−/+^, in cortex and in hippocampus. In wild‐type brains (picture in Supporting Information Figure S3), p‐H3 signals located exclusively in hippocampus (*p *=* *0.005). In Sgo1^−/+^ brains, high expression of p‐H3 in the cortex was observed, hence p‐H3 expression was not limited to hippocampus in Sgo1^−/+^. Percentages of p‐H3‐positive cells in Sgo1^−/+^ cortex were significantly higher compared with those in cortex of age‐matched wild‐type control (*p *=* *0.0003). Although p‐H3 expression in hippocampus was modestly higher in Sgo1^−/+^ compared with wild type, the difference was not significant (*p* = 0.1153). [Asterisk (*): *p *<* *0.05. ns (nonsignificant): *p *>* *0.05]

Dentate Gyrus (DG) and subgranular zone in hippocampus are known to be sites for adult neurogenesis (Bordiuk, Smith, Morin, & Semënov, 2014). Hippocampus is also known to be the site functionally affected by LOAD, leading to the primary LOAD symptom of memory defect. We tested whether amyloid‐β‐and‐p‐H3‐positive cells in Sgo1^−/+^ localize in a particular area (e.g., hippocampus) in the brain. Amyloid‐β‐and‐p‐H3‐positive cells in Sgo1^−/+^ appeared both in the cortex and in the hippocampus (Figure 3a,b). The p‐H3‐positive cell percentages in Sgo1^−/+^ were estimated as 23.96 ∓ 15.32% in the cortex and 16.64 ∓ 6.48% in the hippocampus (Figure 3c). In control wild type (photograph in Supporting Information Figure S3), amyloid‐β‐positive cells were hardly present, and p‐H3‐positive cells were localizing in the hippocampus (10.31 ∓ 6.31%), but not in the cortex (2.33 ∓ 3.82%) (Figure 3c). The data demonstrate that amyloid‐β‐and‐p‐H3‐positive cells characteristically (*p *<* *0.05) appear in the cortex of Sgo1^−/+^, although there is a modest (nonsignificant) increase of p‐H3 in the hippocampus of Sgo1^−/+^ compared with wild type as well.

### 2.5 Differentially expressed genes in Sgo1^−/+^ brain

**Figure 4:**
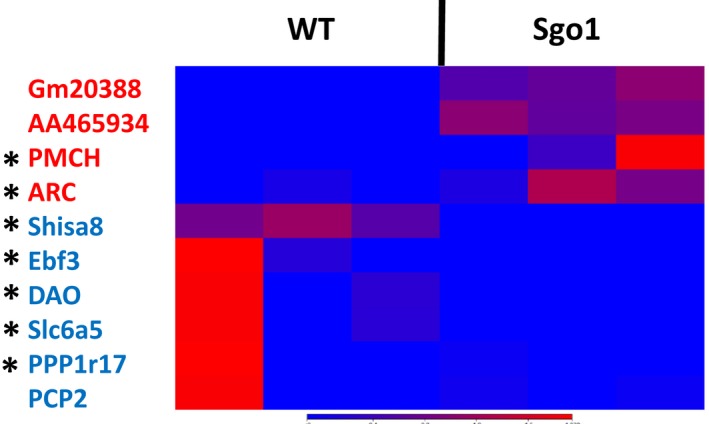
Differential expressions of AD and/or neuronal function‐related genes in the Sgo1^−/+^ brain. Heat map. Blue color indicates lower expression, and red indicates higher expression. Gm20388, AA465934, PMCH, and ARC were more highly expressed in Sgo1^−/+^ mice, while Shisa8, Ebf3, DAO, Slc6a5, PPP1r17, and PCP2 were expressed at lower levels. Genes marked with asterisk had known connections to AD and/or neuronal function (see text)

To elucidate the molecular basis for the AD‐associated brain pathology in Sgo1^−/+^, we used RNA‐seq to compare mRNA expression profiles in 24‐month‐old brains. With *p *<* *0.05 and twofold cutoff, ten genes were identified (Table 1). ARC, PMCH, Gm20388, and AA465934 were overexpressed, while Shisa8, Ebf3, DAO, Slc6a5, PPP1r17, and PCP2 were underexpressed (Figure 4). Among the ten genes, seven had known connections to AD and/or neuronal function.

**Table 1:** Differentially expressed genes in 24‐m‐old Sgo1^−/+^ brain compared with age‐matched wild type (FDR‐*p *<* *0.05, twofold cutoff)

Name	Chromosome	Region	Max group mean	Log_2_ fold change	Fold change	*p*‐Value	FDR *p*‐value
Pmch	10	88091072..88092375	13.84641	4.879923	29.44443198	1.32279E‐06	0.022851667
AA465934	11	83291699..83294632	11.8432	2.27275	4.832432371	7.96492E‐06	0.045865552
Gm20388	8	119910841..124345722	5.28021	1.831427	3.558888713	2.03649E‐06	0.026385744
Arc	15	Complement(74669083.. 7467257 0)	48.70859	1.574903	2.979155502	7.24493E‐06	0.045865552
Shisa8	15	Complement(82206952..8221281 5)	7.245731	−2.05335	−4.150675197	5.81972E‐07	0.015080633
Ebf3	7	Complement(137193673.. 137314 445)	2.481491	−3.54076	−11.63793136	6.98282E‐06	0.045865552
Ppplrl7	6	56017497..56032689	15.43888	−3.71303	−13.11398849	9.40502E‐06	0.048742448
Pcp2	8	Complement(3623371.. 3625545)	24.46027	−4.58608	−24.0185273	4.3674E‐06	0.045268991
Dao	5	114003703..114025682	2.850218	−5.4852	−44.79287015	7.62545E‐06	0.045865552
Slc6a5	7	49910146..49963856	9.454366	−7.18066	−145.0751803	1.41963E‐10	7.35739E‐06

### 2.6 Overexpressed genes in Sgo1^−/+^

Activity‐Regulated Cytoskeleton‐Associated Protein (ARC) [2.97‐fold increase, FDR‐ *p *=* *0.045] is proposed to participate in AD pathogenesis, because ARC is required for activity‐dependent generation of amyloid‐β, and genetic deletion of ARC (−/−) reduces amyloid‐β load in a transgenic mouse model of AD (APP_SWE_;PS1ΔE9) (Wu et al., 2011). ARC upregulation was observed in human AD brain and may directly promote amyloid‐β generation, explaining the pathology in Sgo1^−/+^ mice, at least in part. The pro‐melanin concentrating hormone (PMCH) [29.44‐fold increase, FDR‐*p *=* *0.022] product is processed proteolytically to generate multiple neuropeptides and may regulate energy homeostasis and behaviors such as hunger, reproductive function, and sleep (Mul et al., 2010, 2013). The functions of Gm20388 [3.55‐fold increase, FDR‐*p *=* *0.026] and AA465934 [4.83‐fold increase, FDR‐*p *=* *0.045] remain unclear. The Gm20388 product carries partial homology to Low‐Density Lipoprotein Receptor Class A domain (LDLa) and may be involved in cholesterol metabolism, an AD‐associated pathway.

### 2.7 Underexpressed genes in Sgo1^−/+^

Shisa Family Member 8 (Shisa8) [4.15‐fold decrease, FDR‐*p *=* *0.015] is a Shisa family transmembrane protein, which may be involved in Wnt/FGF signaling or neurotransmitter regulation (Pei & Grishin, 2012). Early B‐cell factor 3 (Ebf3) [11.63‐fold decrease, FDR‐*p *=* *0.045] encodes a transcription factor and may be associated with LOAD (*p *=* *0.03) (Belbin et al., 2011). Mutations in Ebf3 disturb transcriptional profiles and cause intellectual disability, ataxia, and facial dysmorphism in humans (Sleven et al., 2017).

D‐Amino Acid Oxidase (DAO) [44.79‐fold decrease, FDR‐*p *=* *0.045] removes the D‐amino acids that accumulate during aging. DAO degrades D‐serine, a coagonist of the NMDA receptor, and is associated with schizophrenia. A DAO inhibitor Sodium benzoate improved cognitive and overall functions in early‐phase AD patients (Lin et al., 2014). Reduced DAO expression may play a compensating role against the AD‐like pathology in Sgo1^−/+^ model mice.

Solute Carrier Family 6 Member 5 (Slc6a5) [145.07‐fold decrease, FDR‐*p *<* *0.0001] is a sodium‐ and chloride‐dependent glycine neurotransmitter transporter. Slc6a5 physically interacts with Syntaxin 1A, which binds to presenilin 1, mutations in which are linked to familial AD. Slc6a5 is also a component of the NRF2 pathway involved in oxidative stress response. Mutations in Slc6a5 cause hyperekplexia, a neurological disorder with pronounced startle responses and neonatal apnea (Carta et al., 2012).

Protein Phosphatase 1 regulatory subunit 17 (PPP1r17)/G‐substrate [13.11‐fold decrease, FDR‐*p *=* *0.048] is a protein phosphatase inhibitor primarily expressed in Purkinje cells. PPP1r17 is reportedly involved in hypercholesterolemia, long‐term depression, and attenuation in the long‐term adaptation of optokinetic eye movement response (Endo, 2012). Protein phosphatase 1 can dephosphorylate p‐TAU (Liu, Grundke‐Iqbal, Iqbal, & Gong, 2005). Underexpression of PPP1r17 may play a compensatory role against AD‐like pathology via dephosphorylating p‐TAU. PCP2 (Purkinje Cell Protein 2) [24.01‐fold decrease, FDR‐*p *=* *0.045] may function as a modulator for G protein signaling. However, PCP2^−/−^ knockout mice showed no phenotype (Mohn, Feddersen, Nguyen, & Koller, 1997), making elucidation of the function difficult.

## 3 DISCUSSION

Our results suggest that accumulated amyloid‐β originated from p‐H3‐positive prolonged mitotic cells, which later die and leave extracellular deposits including amyloid‐β and p‐TAU that may become seeds for “plaques and tangles.” Cells with accumulated amyloid‐β were specifically observed in Sgo1^−/+^ model mice with intact spindle checkpoint and not in mitotic checkpoint‐defective BubR1^−/+^ model mice. There is supporting evidence suggesting that mitotic cells are involved in amyloid‐β accumulation in human LOAD: (a) Human neurofibrillary tangles colocalized with MPM2 antigens, another mitotic marker (Kondratick & Vandré, 1996); (b) Abnormal Tau phosphorylation of the Alzheimer‐type also occurred during mitosis in human neuroblastoma SY5Y cells overexpressing Tau (Delobel et al., 2002); (c) APP^Thr668^ phosphorylation in mitosis correlated with increased processing of APP to generate Aβ and the C‐terminal fragment of APP (Judge, Hornbeck, Potter, & Padmanabhan, 2011); (d) Although p‐H3 localization is usually limited in chromatin in many other organs, human AD brain showed a cytoplasmic, diffused pattern of p‐H3 (Ogawa et al., 2003), which was recapitulated in the Sgo1^−/+^ mouse brain (Figures 2d and 3a,b). These reports strongly suggest that human LOAD development can be aided by prolonged mitosis, which the Sgo1^−/+^ model recapitulates. Indeed, in human LOAD, models incorporating the critical role of mitotic cells have been proposed, such as a “simple linear model” that states that human AD pathology develops from mitotic cycle‐reentering neurons that later die (Herrup, 2010), and the “two‐hit model” of human LOAD (Zhu, Lee, Perry, & Smith, 2007; Zhu, Raina, Perry, & Smith, 2004) that purports that LOAD development occurs with (a) oxidative stress and (b) mitotic reentry. Although the direct trigger for mitotic cycle reentry in Sgo1^−/+^ model mice remains unclear, studies on roles of cell cycle regulators, such as Cdk5 (Zhang et al., 2008), and on effects of genes identified through RNA‐seq in this study on the cell cycle, are warranted.

The Sgo1^−/+^ haploinsufficient mouse is a model that displays two direct effects of a reduction in Sgo1, both of which lead to prolonged mitosis via the spindle checkpoint: (a) cohesinopathy in mitotic chromosome; and (b) defects in centrosome integrity (Yamada et al., 2012). Whether the AD pathology is caused by cohesinopathy, centrosome defect, or the common consequence that is prolonged mitosis must be distinguished. Cohesinopathy in humans leads to diseases with cancer proneness, developmental malformation, and/or intellectual disability and behavioral issues, such as Cornelia de Lange syndrome or mutations in STAG1 or STAG2 (Kline et al., 2017; Kumar et al., 2015). The symptoms suggest that maintenance of chromosome cohesion may play a role more critical than previously anticipated in brain functions.

Mutations in centrosomal genes are often connected to developmental malformations of the brain, such as autosomal recessive primary microcephaly, microcephalic osteodysplastic primordial dwarfism type II, and Seckel syndrome, in humans (Nigg, Čajánek, & Arquint, 2014). These rare human diseases have not been studied in the context of AD, in part because they are rare yet patients do not survive long, and because mental retardation symptom is difficult to distinguish from cognitive dysfunction of AD. However, the use of corresponding mouse models should provide guidance. At this moment, we do not think Sgo1 is the only target gene to create spontaneous LOAD mouse model. We speculate that functional equivalent of Sgo1 mutation can also create LOAD model. Indeed, functional equivalent of Sgo1 mutation, such as accumulation of aneuploid cells (Bajic et al., 2015; Potter, 1991) or increase in cells reentering mitotic cycle, does occur in human LOAD (Herrup, 2010; Zhu et al., 2004, 2007).

Our results also suggest that prolonged mitosis and/or mitotic spindle checkpoint may have potential as a therapeutic target for AD. Agreeing with this prediction, Flavopiridol, a CDK inhibitor, reversed memory impairment in amyloid‐β‐injected AD mouse model, suggesting the detrimental role of prolonged mitosis in AD (Leggio et al., 2016).

Here, we present evidence that the Sgo1^−/+^ haploinsufficient mouse model displays AD‐like brain pathology at an age equivalent to human old age. The model will allow us to test genetic interactions between known AD‐associated genes (e.g., APOE, ARC) through simple breeding, as well as the influence from environmental, dietary, and other intervention or therapeutic measures. We propose the use of the Sgo1^−/+^ model with pathological assessments as endpoints at this time. Due to the difference in original study aim (i.e., aging‐associated carcinogenesis), whether the mice indicate cognitive and behavioral defects and represent other major hallmarks of human AD have not been tested. However, genes directly or indirectly involved in AD pathology and its modulation (e.g., ARC, DAO, Ebf3, PPP1r17), along with genes that modulate neuronal function and/or behavior (e.g., PMCH, Shisa8, Slc6a5), were identified in the present study, strongly suggesting that Sgo1^−/+^ haploinsufficiency affects the animal's cognitive functions and/or behavior at later ages. Further validation of the model including cognitive function assessment is warranted. Whether the identified genes represent valid AD drug targets is also open for further investigation.

Overall, the model would represent the first genetically defined spontaneous LOAD model once fully validated and would be beneficial for investigating the mechanisms of LOAD pathology development and in translational studies for intervention and therapy.

## 4 EXPERIMENTAL PROCEDURES

### 4.1 Animals

C57BL/6‐based wild‐type (WT), Sgo1^−/+^ (Rao et al., 2016; Yamada et al., 2012, 2015, 2016), and BubR1^−/+^ (Dai et al., 2004; Rao et al., 2005) mice were bred and maintained in a pathogen‐free rodent barrier facility without treatment for 24–25 months (an observational study). Surviving animals were euthanized and organs were collected following our standard operating procedures (Yamada et al., 2012). Animal numbers were as follows: WT, *N *=* *14 (surviving out of 18); Sgo1^−/+^, *N *=* *8 (out of 12); BubR1^−/+^, *N *=* *15 (out of 21). All procedures were approved by the OUHSC Institutional Animal Care and Use Committee. Each brain was split into two hemispheres. One hemisphere was saved in 10% buffered formalin for immunofluorescence, and the other was stored at −80°C after flash freezing in liquid nitrogen for immunoblots and/or RNA‐seq.

### 4.2 Immunoblots

Frozen brain samples (mouse cerebrum including cortex and hippocampus, excluding olfactory bulb, cerebellum, medulla) were extracted in extraction buffer and subjected to immunoblots following our standard protocol (Rao et al., 2016; Yamada et al., 2012). Blots were quantified using imagej 1.43 software (NIH). β‐actin blots were used for loading control and normalization.

### 4.3 Immunofluorescence

Formalin‐fixed brain hemispheres were embedded in paraffin and sectioned onto slides. After deparaffinization, antigen retrieval, sodium borohydride treatment, CuSO_4_ treatment, and blocking, the slides were treated with primary antibodies for 16 hr, then with secondary fluorescent antibodies for 1 hr, followed by brief DAPI staining and sealing with antifade. Sodium borohydride and CuSO_4_ were used to minimize autofluorescence by Shiff‐base and by Lipofuscin, respectively (Schnell, Staines, & Wessendorf, 1999; Spitzer, Sammons, & Price, 2011).

We used multiple antibodies from different vendors, especially for APP and/or amyloid‐β, to ensure accuracy of results. We used the following antibodies: anti‐APP/amyloid‐β [Santa Cruz, sc‐28385], anti‐amyloid‐β (D54D2) [Cell Signaling Technologies (CST), 8243T], anti‐TAU (TAU46) [CST, 4019T], anti‐BACE (D10E5) [CST, 5606T], anti‐APP/amyloid‐β (NAB228) [CST, 2450T], anti‐phospho‐Tau (PhosphoS262) [Antibodies‐online/EnoGene, E011111], anti‐phospho‐Histone H3 (S10) [CST, #9701], anti‐phospho‐Histone H3 (S28) [CST, #9713], anti‐Rabbit Cy5 [Jackson ImmunoResearch, #68551], and anti‐mouse Alexa488 [Invitrogen, A11029]. The slides were observed with an Olympus microscope or a Leica microscope. We used the same image acquisition settings for all samples, so that visualized signal intensity would reflect the difference among samples (e.g., Figure 1a,b).

### 4.4 Quantification of Immunofluorescence

In the experiment in Figure 3 and Supporting Information Figure S3, immunofluorescence samples from wild‐type and Sgo1^−/+^ brains (WT *N* = 3, Sgo1^−/+^ *N* = 3) were photographed (5‐8 sets per animal). In a pictured field, numbers of p‐H3‐positive cells and DAPI‐positive cells were counted, and percentages of p‐H3‐positive cells among all DAPI‐positive cells were calculated. Minimum six fields were analyzed both for cortex and for hippocampus of wild type and of Sgo1^−/+^.

### 4.5 RNA‐seq

Comparative RNA sequencing was performed as in (Rao et al., 2016; Yamada et al., 2016). The total RNA was extracted from frozen brain samples (mouse cerebrum including cortex and hippocampus, excluding olfactory bulb, cerebellum, medulla). The RNA samples were submitted to the OUHSC Laboratory for Bioinformatics core facility for library construction and RNA sequencing with an Illumina MiSeq next‐generation sequencer with each run generating approximately 30 million 2 × 150 bp paired‐end reads. The readouts were analyzed with Strand bioinformatics software (Strand NGS, San Francisco, CA, USA). Illumina MiSeq paired fastq files were aligned in strand ngs software version 2.1 (http://www.strand-ngs.com) using mouse mm10 (UCSC) assembly. The Dec. 2011 Mus musculus assembly (Genome Reference Consortium Mouse Build 38 [GCA_000001635.5]) was produced by the Mouse Genome Reference Consortium (http://genome.ucsc.edu/). Reads were normalized using DESeq. The normalized read counts were log‐transformed and base‐lined to the data set, resulting in normalized signal values. Differential gene expression of the normalized signal values between the control and experimental group was determined using a moderated *t* test, *p *<* *0.05. The differentially expressed gene list was subsequently used for clustering and pathway analysis.

### 4.6 Statistical analysis (RNA‐seq)

We used Student's *t* test to analyze the data. Statistical significance was evaluated by algorithms integral to the aforementioned software. FDR‐adjusted *p* values of <0.05 were considered significant.

### 4.7 Data and materials availability

The RNA‐seq dataset was deposited to the NIH‐GEO database (accession number GSE115185) and will be available there on June 26, 2018. The reagents described in this article are available under a material transfer agreement with University of Oklahoma Health Sciences Center.

## CONFLICT OF INTEREST

The authors declare no conflict of interests.

## AUTHOR CONTRIBUTIONS

C.V. Rao and H.Y. Yamada contributed all aspects of the project. M. Farooqui and Y. Zhang contributed animal maintenance, sample collection, and key data generation. A.S. Asch provided material support and intellectual input.
