# Supplementary figures and images for: Spontaneous development of Alzheimer's disease‐associated brain pathology in a Shugoshin‐1 mouse cohesinopathy model
Source: Aging Cell. 2018 Jun 25;17(4):e12797. doi: 10.1111/acel.12797 (PMC6052391; doi:10.1111/acel.12797)

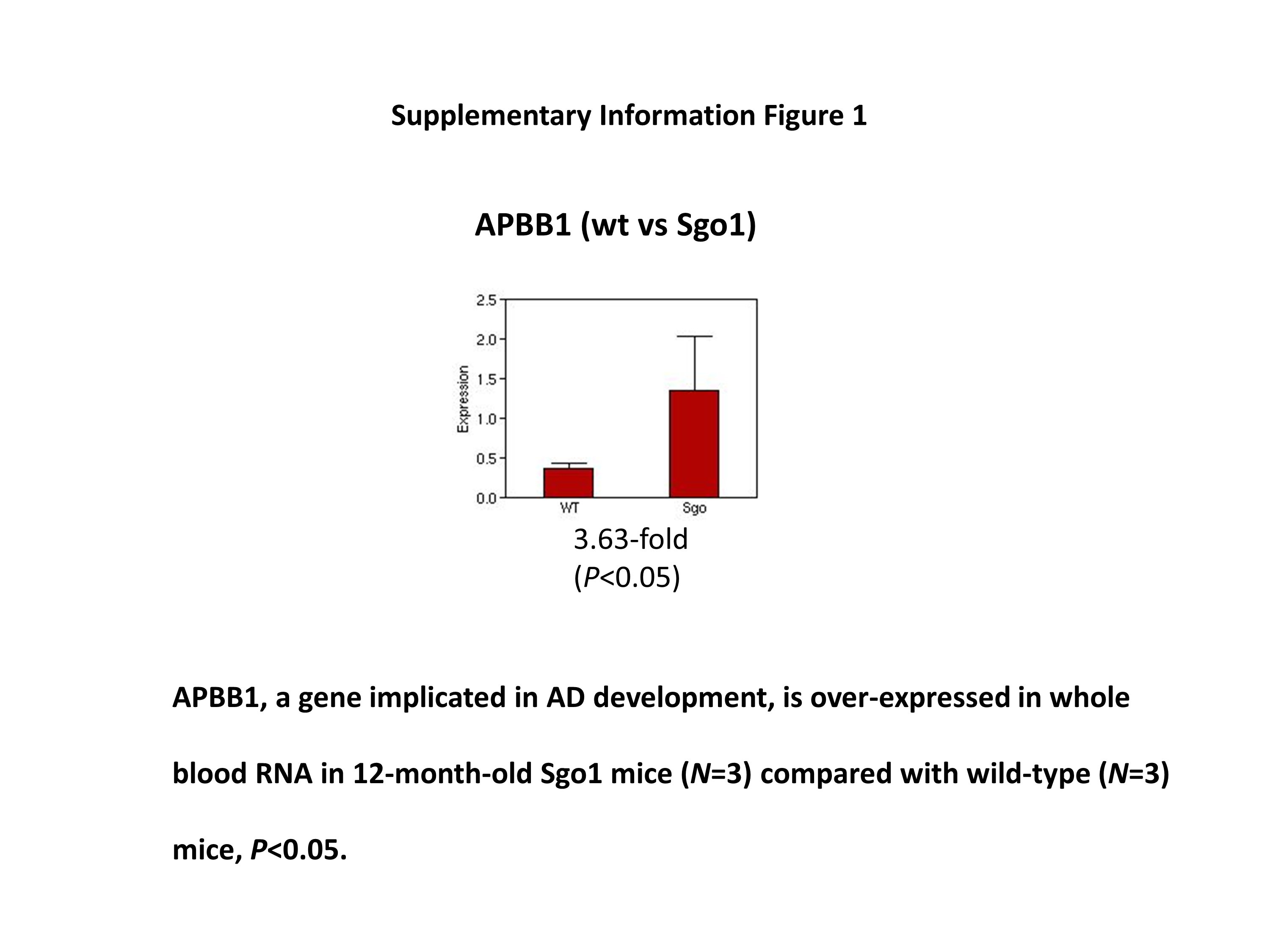

Supplement: Supplementary file 1 [file ACEL-17-na-s001.tif]
